# Identification of candidate genes involved in isoquinoline alkaloids biosynthesis in *Dactylicapnos scandens* by transcriptome analysis

**DOI:** 10.1038/s41598-017-08672-w

**Published:** 2017-08-22

**Authors:** Si Mei He, Wan Ling Song, Kun Cong, Xiao Wang, Yang Dong, Jing Cai, Jia Jin Zhang, Guang Hui Zhang, Jian Li Yang, Sheng Chao Yang, Wei Fan

**Affiliations:** 1 State Key Laboratory of Conservation and Utilization of Bio-resources in Yunnan, National& Local Joint Engineering Research Center on Gemplasm Innovation & Utilization of Chinese Medicinal Materials in Southwest China, Yunnan Agricultural University, Kunming, 650201 China; 2 State Key Laboratory of Genetic Resources and Evolution, Kunming Institute of Zoology, Chinese Academy of Sciences, Kunming, 650223 China; 3 Province Key Laboratory, Biological Big Data College, Yunan Agricultural University, Kunming, 650201 China; 4 State Key Laboratory of Quality Research in Chinese Medicine, Institute of Chinese Medical Sciences, University of Macau, Macau, 999078 China; 5 Graduate School of the Chinese Academy of Sciences, Beijing, 100049 China; 6 State Key Laboratory of Plant Physiology and Biochemistry, College of Life Sciences, Zhejiang University, Hangzhou, 310058 China

## Abstract

*Dactylicapnos scandens* (D. Don) Hutch (Papaveraceae) is a well-known traditional Chinese herb used for treatment of hypertension, inflammation, bleeding and pain for centuries. Although the major bioactive components in this herb are considered as isoquinoline alkaloids (IQAs), little is known about molecular basis of their biosynthesis. Here, we carried out transcriptomic analysis of roots, leaves and stems of *D*. *scandens*, and obtained a total of 96,741 unigenes. Based on gene expression and phylogenetic relationship, we proposed the biosynthetic pathways of isocorydine, corydine, glaucine and sinomenine, and identified 67 unigenes encoding enzymes potentially involved in biosynthesis of IQAs in *D*. *scanden*s. High performance liquid chromatography analysis demonstrated that while isocorydine is the most abundant IQA in *D*. *scanden*s, the last *O*-methylation biosynthesis step remains unclear. Further enzyme activity assay, for the first time, characterized a gene encoding *O*- methyltransferase (DsOMT), which catalyzes *O*-methylation at C7 of (*S*)-corytuberine to form isocorydine. We also identified candidate transcription factor genes belonging to WRKY and bHLH families that may be involved in the regulation of IQAs biosynthesis. Taken together, we first provided valuable genetic information for *D*. *scandens*, shedding light on candidate genes involved in IQA biosynthesis, which will be critical for further gene functional characterization.

## Introduction

Alkaloids are a large and diverse group of nitrogenous secondary metabolites that account for approximately 20% of plant species. Alkaloids mainly include isoquinoline alkaloids(IQAs), quinoline alkaloids, pyrrolidine alkaloids, and indole alkaloids, among which IQAs are the largest and most important alkaloids that specifically present in Magnoliaceae, Ranunculaceae, Papaveraceae and Berberidaceae. Current studies on IQAs with respect to separation of chemical constituents and pharmacodynamics functions showed that IQAs play key roles in anti-inflammatory and analgesia, thereby serving as the analgesics morphine and codeine, the antitumor agent noscapine, the muscle relaxant papaverine, and the antimicrobial agents sanguinarine and berberine^1–4^. However, the molecular mechanisms catalyzing and regulating IQAs biosynthesis in plants are still unclear because of the structural diversity of IQAs present in different plant species.

The biosynthesis of IQAs begins with conversion of L-tyrosine to dopamine and 4-hydroxyphenylacetaldehyde, which are then condensed to (*S*)-norcoclaurine by (*S*)-norcoclaurine synthase (NCS)^5–7^. Three methyltransferases [(*S*)-norcoclaurine 6-*O*-methyltransferase (6OMT), (*S*)-coclaurine-*N*-methyltransferase (CNMT) and (*S*)-3′-hydroxy-*N*-methylcoclaurine-4′-*O*-methyltransferase (4′OMT)] and one hydroxylase [(*S*)-*N*-methylcoclaurine 3′-hydroxylase (NMCH)] are involved in catalyzing the conversion of (*S*)-norcoclaurine to (*S*)-reticuline which has been considered as a common intermediate of many IQAs^8–11^. At present, biosynthetic pathways for several kinds of IQAs have been widely reported in a number of plant species, such as berberine in *Coptis japonica*, sanguinarine in *Eschscholzia californica* and morphine in *Papaver somniferum*, and many enzymes involved in IQAs biosynthesis in these plants have been characterized^12–14^. In the IQAs biosynthesis, many steps involve oxidation reaction catalyzed by cytochrome P450s (P450s) and *O*-methylation process catalyzed by OMTs family, which participate in synthesis of intermediate products (*S*)-reticuline, and subsequent multistep transformations to form different end products. P450 play a key role in oxidative reactions, including methylenedioxy bridge formation, intramolecular C–C phenol-coupling and intermolecular C–O phenol-coupling reactions^15–17^. *CYP719B1* encoding salutaridine synthase in *P*. *somniferum* has been characterized to catalyze C-C phenol-coupling reaction in morphine biosynthesis^18^. Three *CYP719A* genes encoding canadine synthase, cheilanthifoline synthase and stylopine synthase, respectively, in *C*. *japonica* and *E*. *californica* catalyze methylenedioxy bridge-forming reactions in IQAs biosynthesis^19, 20^. Recently, it has been reported that (*S*)-*cis*-*N*-methylstylopine 14-hydroxylase (MSH), a member of the CYP82N subfamily of P450, catalyzes C–O couplings in sanguinarine biosynthesis from *P*. *somniferum* ^21^. *O*-methylation involves the transfer of the methyl group of SAM to the hydroxyl group of an acceptor molecule, resulting in formation of a methyl ether derivative and S-adenosyl-L-homocysteine. *O*-methylation of different C sites is catalyzed by different types of OMTs. Many OMTs in several plants have been characterized, including (1) 6OMT from *C. japonica* ^8^ and *P. somniferum* ^22^; (2) 4′OMT from *C*. *japonica* ^8^, *P*. *somniferum* ^23^, and *E. californica* ^24^; (3) ﻿norreticuline 7-*O*-methyltransferase (N7OMT) from *P*. *somniferum* ^25^; (4) reticuline 7-*O*-methyltransferase (7OMT) from *P*. *somniferum* ^22^; (5) scoulerine-9-*O*-methyltransferase (SOMT) from *C*. *japonica* ^26^ and *P*. *somniferum* ^27^; and (6) columbamine *O*-methyltransferase (CoOMT) from *C*. *japonica* ^28^. Recently, three *O*-methyltransferases, designated as SOMT1, SOMT2, and SOMT3 have been reported to be involved in noscapine biosynthesis in *P*. *somniferum*. SOMT1 is able to sequentially 9- and 2-*O*-methylate (*S*)-scoulerine, yielding (*S*)-tetrahydropalmatine, and also sequentially 3′- and 7-*O*-methylate both (*S*)-norreticuline and (*S*)-reticuline with relatively high substrate affinity, yielding (*S*)-tetrahydropapaverine and (*S*)-laudanosine, respectively. In contrast, SOMT2 and SOMT3 showed strict substrate specificity and regiospecificity as 9-*O*-methyltransferases targeting (*S*)-scoulerine^27^. Although IQAs biosynthesis such as morphine, sanguinarine and berberine has been well studied, other IQAs biosynthesis such as isocorydine and sinomenine is not well understood.

*Dactylicapnos scandens* (D. Don) Hutch, mainly distributing in northwestern India, Thailand, Tibet Autonomous Region and Yunnan Province in China, is a tuberous rooted perennial herb belonging to Papaveraceae^29, 30^. As a famous traditional Chinese medicine, it is a popular Bai folk medicine and has been used for treatment of inflammation, hypertension, bleeding and pain^31^. The main bioactive constituents of *D*. *scandens* are IQAs, including isocorydine, corydine, glaucine, sinomenine, protopine and magnoflorine^32–34^. Chemical investigation of *D*. *scandens* revealed that isocorydine was the most abundant^34, 35^, suggesting that it is a very good plant material to study the biosynthetic pathway of isocorydine. However, the genome of *D*. *scandens* has not been sequenced and genetic resources are scarce. In this study, we performed the transcriptomes of roots, leaves and stems of *D*. *scandens* using the Illumina HiSeq. 2000 sequencing platform. Based on previous reports^12, 36–38^ and current transcriptomic analysis, we proposed the integrated biosynthetic pathways for isocorydine, corydine, glaucine and sinomenine, and identified probably candidate genes that encode enzymes and transcription factors (TFs) controlling the IQAs biosynthesis in *D*. *scandens*. Furthermore, we characterized a candidate gene encoding OMT protein, *DsOMT*, which catalyzes *O*-methylation at C7 of (*S*)-corytuberine to form isocorydine.

## Results and Discussion

### Illumina sequencing and *de novo* assembly

**Figure 1:**
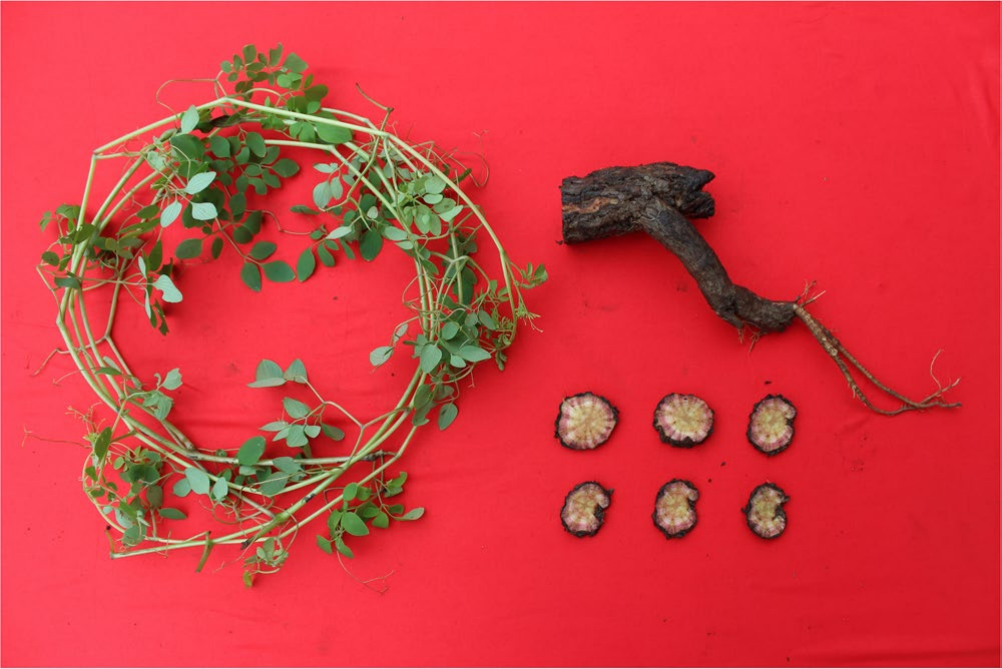
Roots, leaves and stems of *D*. *scandens*. Wild seedlings of *D*. *scandens* were transplanted into the field for 1.5 years.

To obtain a comprehensive understanding of *D*. *scandens* transcriptome, the cDNA libraries were constructed from total RNA of *D*. *scandens* roots, leaves and stems, respectively (Fig. 1). Three biological replications for each tissue were sequenced using the Illumina HiSeq. 2000 sequencing platform. After filtering out adaptor sequences, ambiguous reads and low-quality reads (Q20 < 20), a total of 3.7, 3.9 and 3.6 Gb of high-quality reads from roots, leaves and stems, respectively, were generated (Supplementary Table S1). The high-quality reads obtained in this study were deposited in the NCBI SRA database (accession number: SRA480383).

Because genome information is unavailable for *D*. *scandens*, all clean reads were *de novo* assembled using Trinity software^39^, with optimized k-mer length of 25. We obtained a total of 96,741 unigenes with sequence length ranged from 201 to 17,943 bp, and a total length of 114,753,746 bp. The average length of all unigenes is 905 bp, and there are 34,151 unigenes (26.94%) longer than 1,000 bp. The coding DNA sequences (CDS) from all *D*. *scandens* unigene sequences were also detected and a total of 30,190 CDSs were obtained. Among them, 26.93% unigenes have CDS longer than 1,000 bp. The summary of sequencing and assembly results was shown in Table 1, and the length distribution of the transcripts, unigenes and CDS were shown in Supplementary Fig. S1.

**Table 1:** Summary of Illumina paired-end sequencing and assembly for *D*. *scandens*.

Database	Number	Total length (bp)
Number of transcripts	126,748	114,753,746
Average length of transcripts	905 bp	
Max length of transcripts	17,943 bp	
Min length of transcripts	201 bp	
Transcript size N50	1,738 bp	
Number of unigenes	96,741	68,493,388
Average length of unigenes	708 bp	
Max length of unigenes	17,943 bp	
Min length of unigenes	201 bp	
Unigene size N50	1,203 bp	

### Functional annotation

To annotate these assembled unigenes as many as possible, sequences were searched against seven public protein databases: NCBI nucleotide (NT), NCBI non-redundant protein (NR), SwissProt protein, Gene Ontology (GO), euKaryotic Ortholog Groups (KOG), Kyoto Encyclopedia of Genes and Genomes (KEGG) and Protein family (PFAM). A total of 37,783 unigenes (39.05%) were annotated in the public databases and, of these, 5317 unigenes were annotated in all databases. There were 28,557 unigenes (29.51%) matched in the NR database, and 22,315 unigenes (23.06%) matched with known proteins in the SwissProt database. A total of 23,412 unigenes (24.20%) matched to the GO database and 11,545 unigenes (11.93%) matched to the KOG. The number of unigenes matched to the NT, KEGG and PFAM databases was 20,673 (21.36%), 10,516 (10.87%) and 23,149 (23.92%), respectively (Table 2).

**Table 2:** Summary of the annotation percentage of *D*. *scandens* compared to public databases.

Database	Number of unigenes	Annotation percentage (%)
NR	28,557	29.51
Swiss Prot	22,315	23.06
GO	23,412	24.20
KOG	11,545	11.93
NT	20,673	21.36
KO	10,516	10.87
PFAM	23,149	23.92
Annotated in all databases	5,317	5.49
Annotated in at least one database	37,783	39.05
Total unigenes	96,741	100.00

For GO analyses, 415,147 unigenes were classified into three classes, including biological processes (274,720 unigenes), cellular components (86,318 unigenes), and molecular functions (54,109 unigenes) (Supplementary Fig. S2). There were 12,915 unigenes assigned to KOG classifications, which were divided into 25 specific categories. Predominantly unigenes were found in the category of general functional prediction that is associated with only basic physiological and metabolic functions (2,038, 15.78), whereas unigenes belonging to category of cell motility was the smallest group, with only four unigenes (0.03%) (Supplementary Fig. S3). The KEGG pathway-based analysis is helpful for understanding the biological functions and interactions of genes. A total of 10,782 unigenes had significant matches in the KEGG database and were assigned to 129 biological pathways. The category with the largest number of unigenes was metabolism that includes the biosynthesis of other secondary metabolites (367, 6.32%). Among them, predominantly category was phenylpropanoid biosynthesis (166 unigenes, 45.23%), followed by IQAs biosynthesis (44, 11.99%) and other biosynthesis (Supplementary Fig. S4).

### Quantitative analysis of three major IQAs in *D*. *scandens*

**Figure 2:**
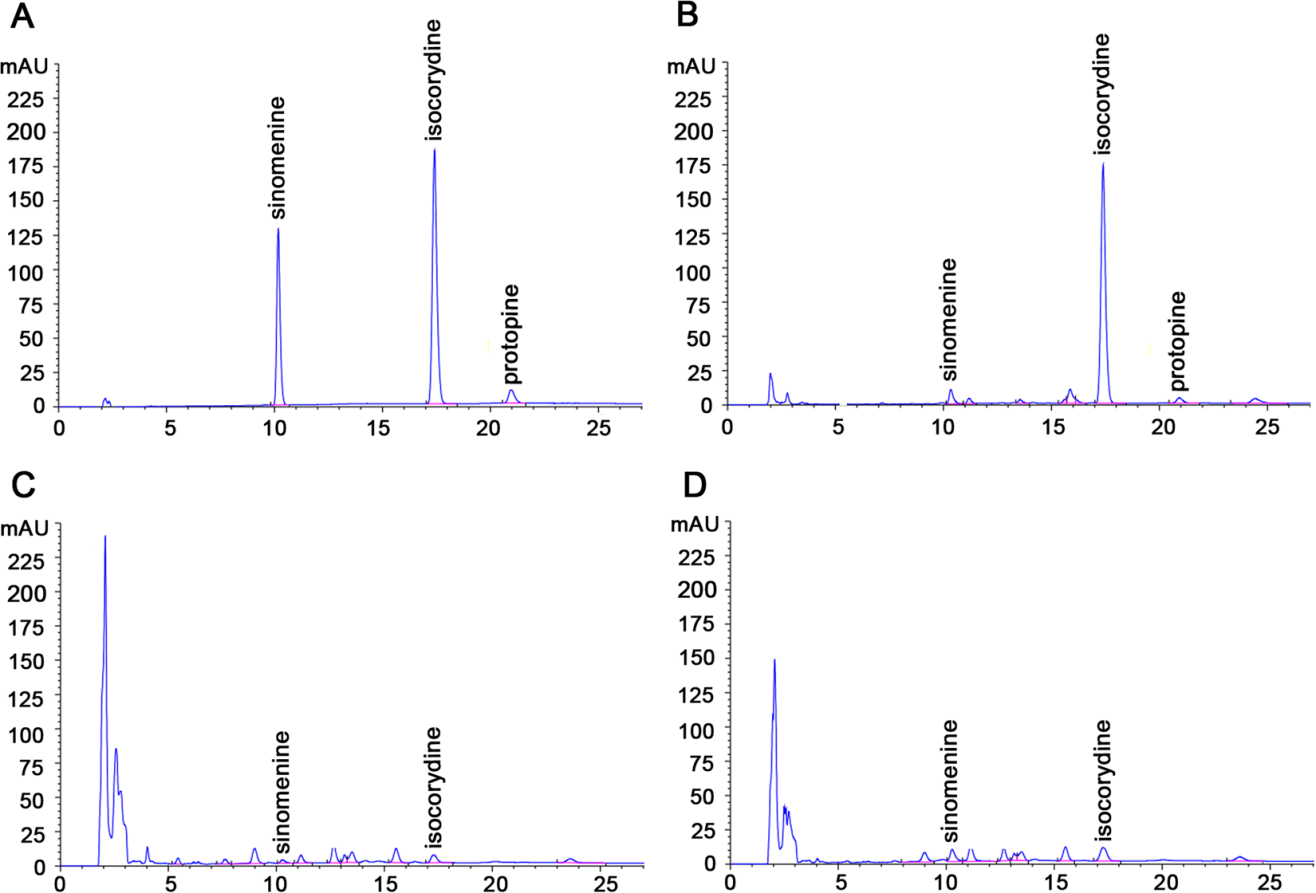
Chromatograms of three major IQAs in roots, leaves and stems of *D*. *scandens*. (**A**) HPLC chromatograms of sinomenine, isocorydine and protopine standards. HPLC chromatograms of sinomenine, isocorydine and protopine in *D*. *scandens* (**B**) roots, (**C**) leaves and (**D**) stems.

According to previous reports, isocorydine, sinomenine and protopine are the main IQA components of *D*. *scandens* ^32–34^. Herein, the content of these major IQAs in roots, leaves and stems of *D*. *scandens* was quantified by high performance liquid chromatography (HPLC). Compared to authentic standards, the contents of isocorydine, sinomenine and protopine in roots were 6.074, 0.822 and 1.824%, respectively (Fig. 2A,B), indicating that *D*. *scandens* roots are abundant in isocorydine. By contrast, the contents of isocorydine, sinomenine and protopine in leaves and stems were very low and almost undetectable (Fig. 2C, D). These results were in accordance with roots being the main medicinal part of the plant. Isocorydine, an aporphine alkaloid with one free hydroxyl group, can inhibit cell proliferation by inducing G2/M cell cycle arrest and apoptosis and target the drug-resistant cellular side population through PDCD4-related apoptosis in hepatocellular carcinoma (HCC)^40–42^, thus can be served as a potential antitumor agent in HCC. This indicates that isocorydine is a kind of pharmaceutically valuable IQAs. However, the molecular mechanisms involved in isocorydine biosynthesis remain unclear.

### Candidate genes encoding enzymes involved in IQAs biosynthesis

**Figure 3:**
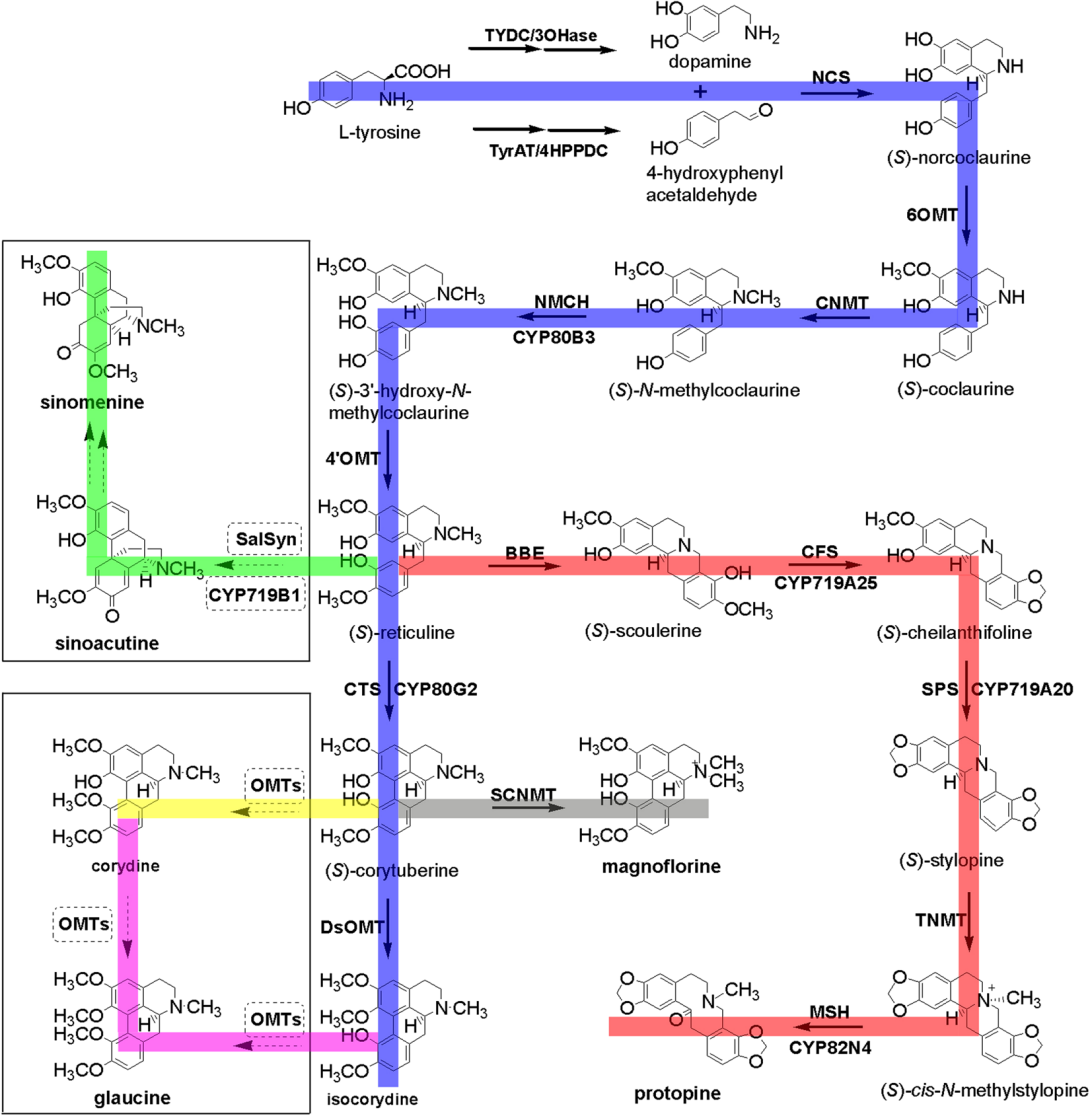
Putative pathways for IQAs biosynthesis in *D*. *scandens*. Enzymes found in this study are boxed. Abbreviations: TyrAT, L-tyrosine aminotransferase; 4HPPDC, 4-hydroxyphenylpuruvate decarboxylase; TYDC, tyrosine decarboxylase; 3OHase, tyrosine/tyramine 3-hydroxylase; NCS, (*S*)-norcoclaurine synthase; 6OMT, norcoclaurine 6-*O*-methyltransferase; CNMT, (*S*)-coclaurine N-methyltransferase; NMCH, *N*-methylcoclaurine 3′-hydroxylase; 4′OMT, 3′-hydroxy-*N*-methylcoclaurine 4′-*O*-methyltransferase; BBE, berberine bridge enzyme; CFS, (*S*)-cheilanthifoline synthase; SPS, (*S*)-stylopine synthase; TNMT, (*S*)-tetrahydroprotoberberine *N*-methyltransferase; MSH, (*S*)-*cis*-*N*-methylstylopine 14-hydroxylase; CTS, corytuberine synthase; SCNMT, (*S*)-corytuberine-*N*-methyltransferase; SalSyn, salutaridine synthase.

We focused on the discovery of genes involved in IQAs biosynthesis, which begins with the condensation of two L-tyrosine derivatives: 4-hydroxyphenylacetaldehyde and dopamine^43, 44^. Through a series of enzymatic reactions, (*S*)-reticuline is synthesized, which acts as the central intermediate and is diverted to different branches for biosynthesis of different types of IQAs^45^. In *D*. *scandens*, the major active constituents are isocorydine, corydine, glaucine, sinomenine, protopine and magnoflorine^32–34^. To date, although the protopine and magnoflorine pathways have been characterized in other plant sepcies^17, 46^, pathways of isocorydine, corydine, glaucine and sinomenine have not been determined. Therefore, we proposed their biosynthesis pathways based on previous reports and present transcriptome data (Fig. 3).

**Figure 4:**
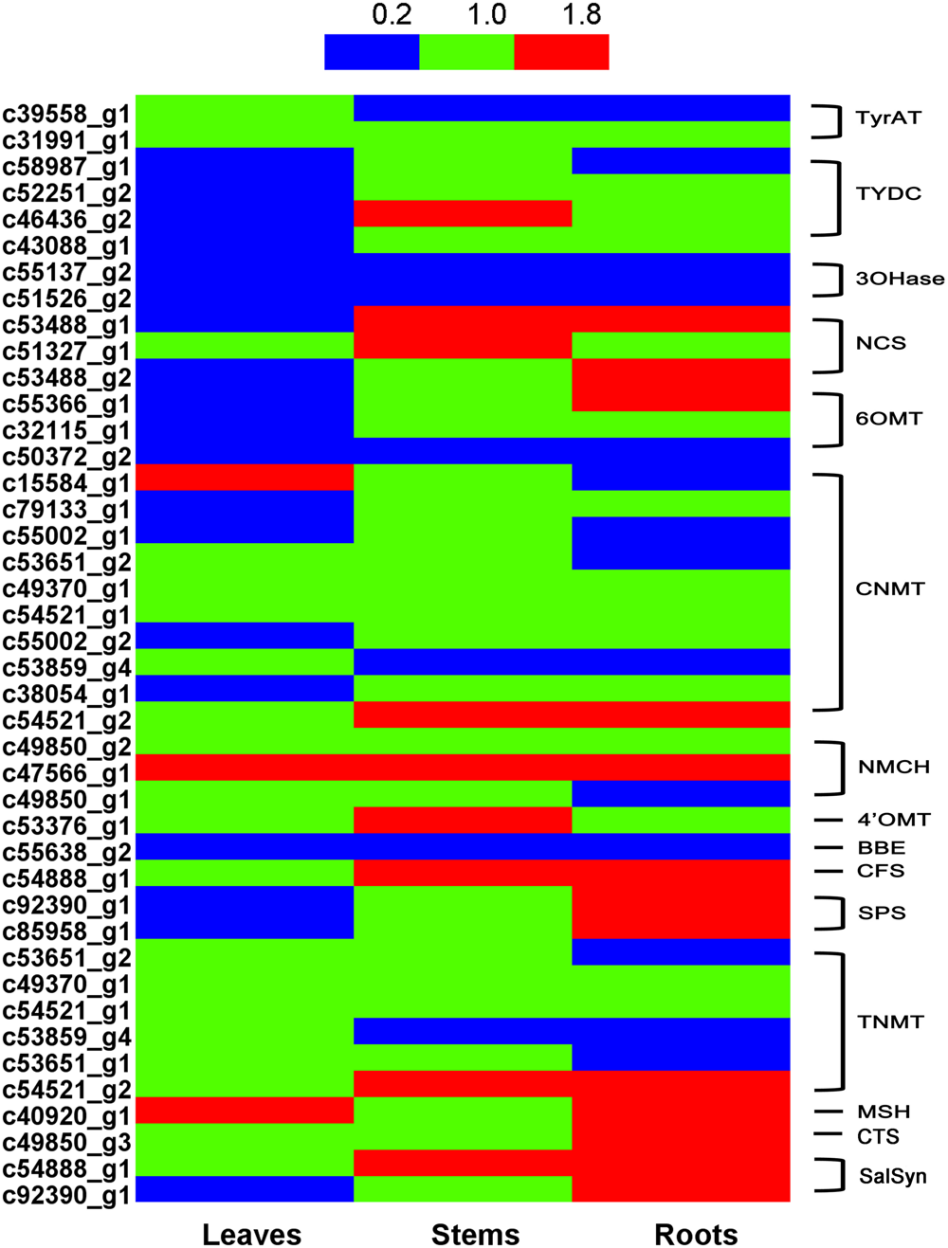
RPKM values of candidate unigenes involved in the biosynthesis of IQAs in *D*. *scandens*. Expression levels of candidate unigenes involved in the biosynthesis of IQAs in *D*. *scandens* leaves, stems and roots.

Based on the KEGG pathway assignment, we discovered the transcripts encoding all the known enzymes for (*S*)-reticuline biosynthesis from our Illumina dataset, which include L-tyrosine aminotransferase (TyrAT), tyrosine decarboxylase (TYDC), tyrosine/tyramine 3-hydroxylase (3OHase), NCS, 6OMT, CNMT, NMCH and 4′OMT. Protopine and magnoflorine biosynthesis pathways have previously been depicted as follows: (*S*)-scoulerine is formed from (*S*)-reticuline by berberine bridge enzyme (BBE)^47, 48^. The conversion of (*S*)-scoulerine to protopine begins with the formation of two methylenedioxy bridges by cheilanthifoline synthase (CFS) and stylopine synthase (SPS), both are members of the CYP719 family, forming (*S*)-stylopine. CFS and SPS have been isolated and characterized from *E*. *californica* ^16, 49^ and Mexican prickly poppy (*Argemone mexicana*)^50^. Subsequent *N*-methylation of (*S*)-stylopine by tetrahydroprotoberberine -*N*-methyltransferase (TNMT)^51^ yields (*S*)-*N*-methylstylopine, which is converted by (*S*)-*cis*-*N*-methylstylopine 14-hydroxylase (MSH) to protopine^21^. In magnoflorine biosynthesis, (*S*)-reticuline is first oxidized to (*S*)-corytuberine by (*S*)-corytuberine synthase (CTS) and, subsequently, (*S*)-corytuberine-*N*-methyltransferase (SCNMT) converts (*S*)-corytuberine to magnoflorine^46^ (Table 3; Supplementary Table 2; Fig. 3). Furthermore, 67 unigenes encoding enzymes involved in IQAs biosynthesis were used to detect their expression levels in different tissues based on reads per kilobase of transcript per million reads mapped (RPKM) values. The results indicated that most genes showed higher expression in roots than leaves or stems, especially for those genes located at downstream of protopine and isocorydine biosynthesis such as *CFS*, *SPS*, *TNMT*, *MSH* and *CTS* (Fig. 4). This is basically consistent with the higher content of IQAs in the roots of *D*. *scandens* (Fig. 2).

**Table 3:** Unigenes involved in biosynthesis of isoquinoline alkaloid.

Gene name	EC number	Unigene numbers
TyrAT, L-tyrosine aminotransferase	2.6.1.5	3
4HPPDC, 4-hydroxyphenylpuruvate decarboxylase	4.1.1.80	0
TYDC, tyrosine decarboxylase	4.1.1.25	4
3OHase, tyrosine/tyramine 3-hydroxylase	1.14.16.2	4
NCS, (*S*)-norcoclaurine synthase	4.2.1.78	3
6OMT, norcoclaurine 6-*O*-methyltransferase	2.1.1.128	10
CNMT, (*S*)-coclaurine *N*-methyltransferase	2.1.1.140	12
NMCH, *N*-methylcoclaurine 3′-hydroxylase	1.14.13.71	4
4′OMT, 3′-hydroxy-*N*-methylcoclaurine-4′-*O*-methyltransferase	2.1.1.116	1
BBE, berberine bridge enzyme	1.21.3.3	1
CFS, (*S*)-cheilanthifoline synthase	1.14.21.2	1
SPS, (*S*)-stylopine synthase	1.14.21.1	2
TNMT, (*S*)-tetrahydroprotoberberine N-methyltransferase	2.1.1.122	11
MSH, (*S*)-*cis* -*N*-methylstylopine 14-hydroxylase	1.14.-.-	1
CTS, corytuberine synthase	1.14.13.21	1
SCNMT, (*S*)-corytuberine-*N*-methyltransferase	2.1.1.-	0

**Figure 5:**
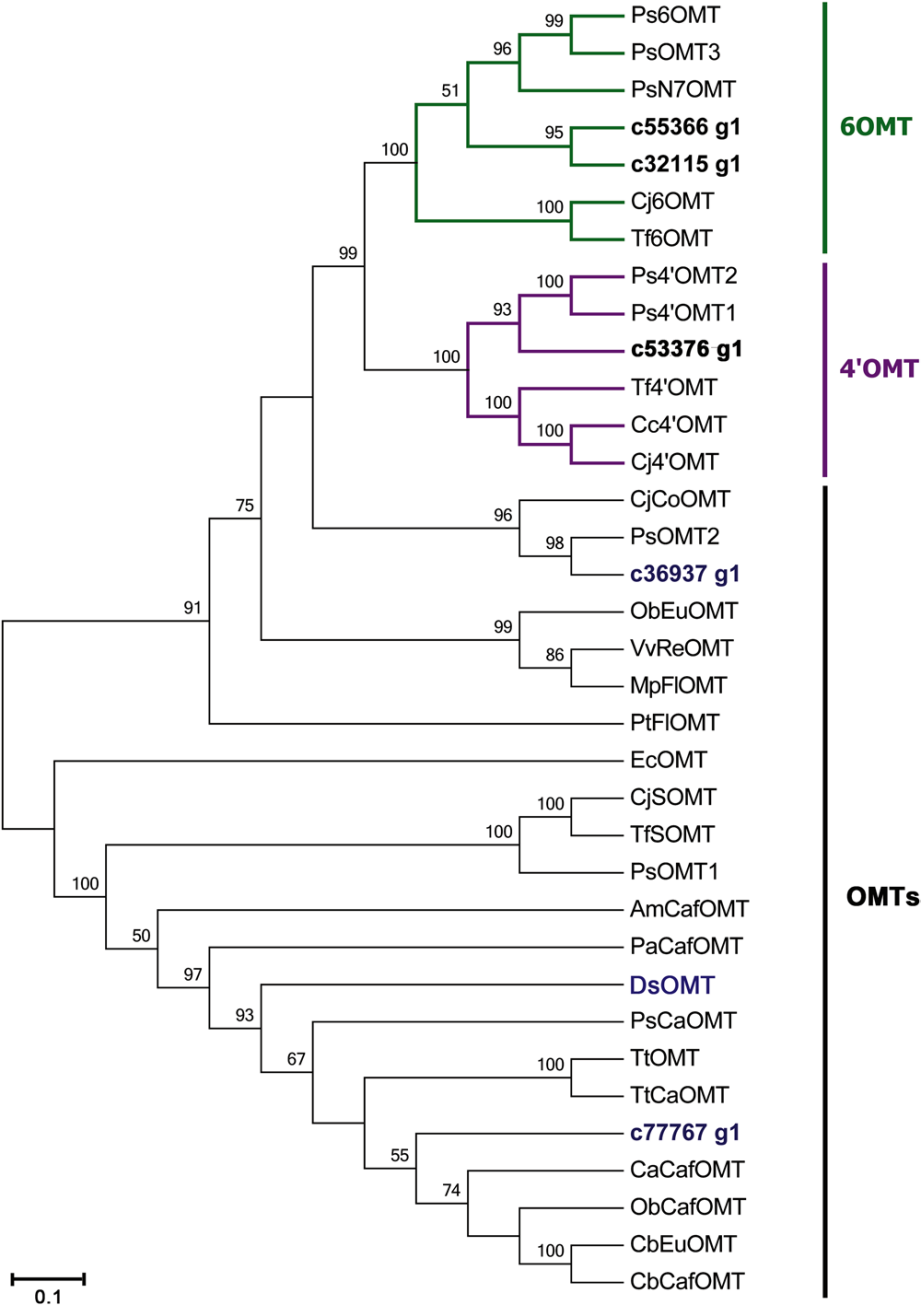
Phylogenetic tree of OMTs. Phylogenetic tree was constructed based on the deduced amino acid sequences for the *D*. *scandens* OMTs (bold letters) and other plant OMTs. Abbreviations and GenBank accession numbers for the sequences used are as follows: EcOMT, putative *Eschscholzia californica* OMT (ACO90220.1); CjCoOMT, *Coptis japonica* columbamine OMT (Q8H9A8.1); TtOMT, *Thalictrum tuberosum* catechol OMT (AAD29845.1); TtCaOMT, *T*. *tuberosum* catechol OMT (AAD29843.1); PsCaOMT, *P*. *somniferum* catechol OMT (AAQ01670.1); PsN7OMT, *P*. *somniferum* norreticuline 7OMT (ACN88562.1); Ps6OMT, *P*. *somniferum* norcoclaurine 6OMT (AAP45315.1); Ps4′OMT2, *P*. *somniferum* 3′-hydroxy-N-methylcoclaurine 4′OMT2 (AAP45314.1); Ps4′OMT1, *P*. *somniferum* 3′-hydroxy-N-methylcoclaurine 4′OMT1 (AAP45314.1); Cc4′OMT, *Coptis chinensis* 3′-hydroxy-N-methylcoclaurine 4′OMT (ABY75613.1); Cj4′OMT, *C*. *japonica* 3′-hydroxy-N-methylcoclaurine 4′OMT (Q9LEL5.1); Tf4′OMT, *T*. *flavum* 3′-hydroxy-N-methylcoclaurine 4′OMT (AAU20768.1); Cj6OMT, *C*. *japonica* norcoclaurine 6OMT (Q9LEL6.1); Tf6OMT, *T*. *flavum* norcoclaurine 6OMT (AAU20765.1); VvReOMT, *Vitis vinifera* resveratrol OMT (CAQ76879.1); PtFlOMT, *Populus trichocarpa* flavonoid OMT predicted protein (XP_002312933.1); CjSOMT, *C*. *japonica* scoulerine 9OMT (Q39522.1); TfSOMT, *T*. *flavum* scoulerine 9OMT(AAU20770.1); PaCafOMT, *Picea abies* caffeate OMT (CAI30878.1); CaCafOMT, *Capsicum annuum* caffeate OMT (AAG43822.1); PsOMT1, *P*. *somniferum* SOMT1 (JN185323); PsOMT2, *P*. *somniferum* SOMT2 (JN185324); PsOMT3, *P*. *somniferum* SOMT3 (JN185325); ObEuOMT, *Ocimum basilicum* eugenol OMT (AAL30424.1); MpFlOMT, *Mentha X piperita* flavonoid 8OMT (AAR09600.1); ObCafOMT, *O*. *basilicum* caffeate OMT (AAD38189.1); CbEuOMT, *Clarkia breweri* (iso)eugenol OMT (AAC01533.1); CbCafOMT, *C*. *breweri* caffeate OMT (O23760.1); and AmCafOMT, *Ammi majus* caffeate OMT (AAR24095.1).

In the biosynthetic pathway of isocorydine, corydine and glaucine, we proposed that the last *O*-methylation reactions were catalyzed by OMTs by affecting different hydroxyl groups. In our dataset, we obtained 10 and 1 unigenes encoding 6OMT and 4′OMT, respectively. Unigenes c36937_g1, c77767_g1, DsOMT, c55366_g1, c32115_g1 and c53376_g1 were highly similar to *P*. *somniferum* PsOMT1, PsOMT2 and PsOMT3, respectively^27^. To characterize the evolutionary relationships between OMTs from *D*. *scandens* and known OMTs from other plant species, Neighbor-Joining tree was constructed. As shown in Fig. 5, unigenes c55366_g1 and c32115_g1 had high homology with 6OMT, while c53376_g1 had high homology with 4′OMT. Notably, we also found that c36937_g1, c77767_g1 and c47357_g1 were not clustered with the known OMTs, suggesting that they may have distinct roles in IAQs biosynthesis, which requires further study.

**Figure 6:**
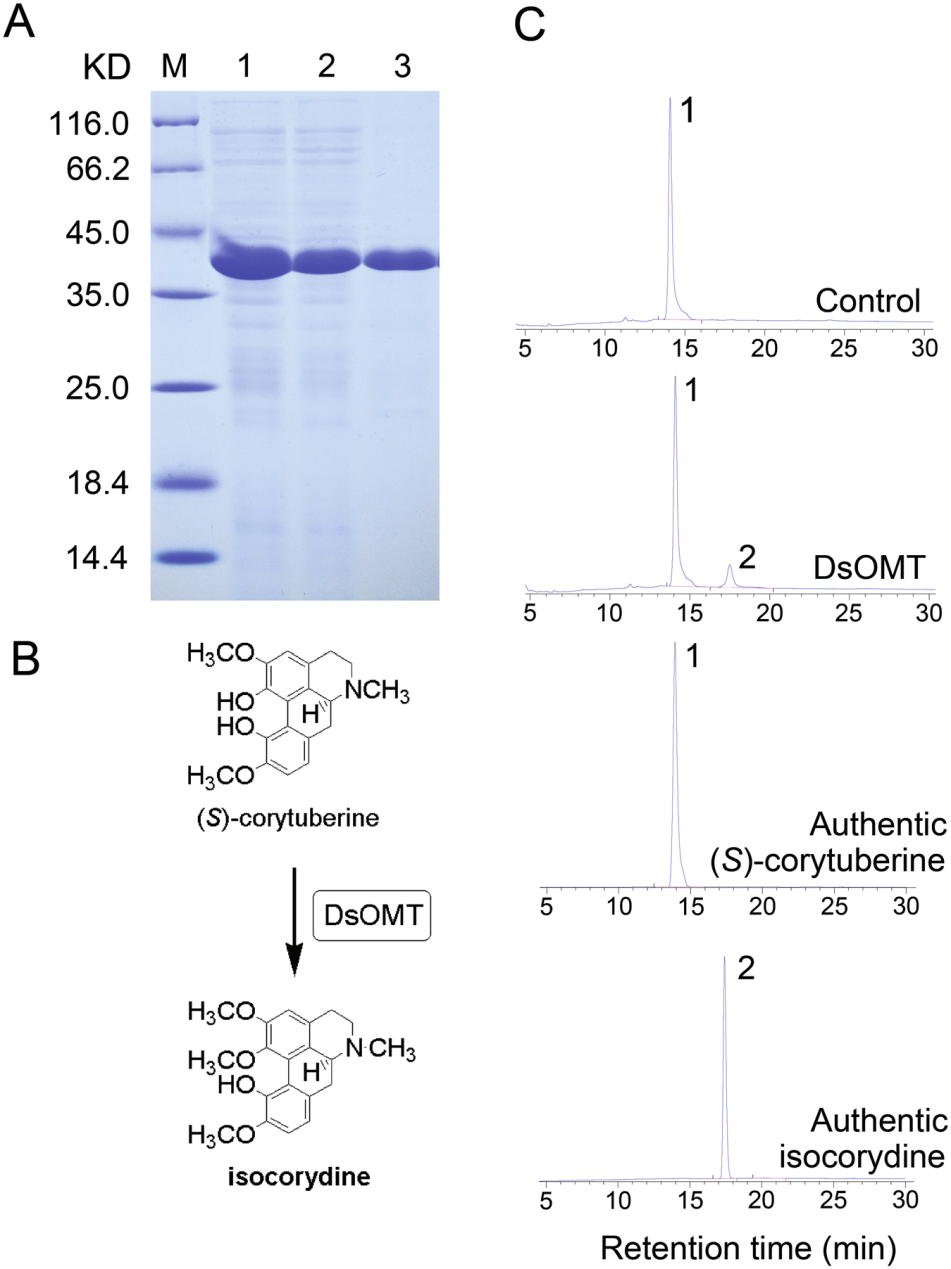
HPLC analysis of *O*-methylation activity of recombinant DsOMT on (*S*)-corytuberine. (**A**) His-tag purified recombinant DsOMT on 12% SDS-PAGE gel, Lane M: Protein Marker, Lane 1: Un-purified, Lane 2: Flow through, Lane 3: Elution; (**B**) verified reaction equation; (**C**) (*S*)-corytuberine with denatured purified DsOMT proteins (control) and *in vitro* assay product of (*S*)-corytuberine with DsOMT; Product was identified using authentic standards, 1. (*S*)-corytuberine; 2. isocorydine.

Previous studies have confirmed the upstream biosynthesis pathway of isocorydine, and the corresponding enzymes have also been characterized^36, 37^. However, the last *O*-methylation step of isocorydine biosynthesis had not been elucidated yet. Here, we supplemented and perfected the isocorydine biosynthesis pathways because it is the most abundant in *D*. *scandens* (Fig. 2). By comparing molecular structure, we found isocorydine was formed by *O*-methylation at C7 on (*S*)-corytuberine. Thus, three functionally unknown OMT unigenes, c36937_g1, c77767_g1 and c47357_g1 (designated as *DsOMT*) were selected to study their function by *in vitro* enzyme activity. The results showed that DsOMT was able to *O-*methylate at C7 on (S)-corytuberine yielding isocorydine (Fig. 6). Therefore, we first established a complete biosynthesis pathway for isocorydine, which will be of potential significance for further understanding the molecular mechanisms of IQAs biosynthesis in plants.

### Identification of transcription factors potentially involved in IQAs biosynthesis

**Figure 7:**
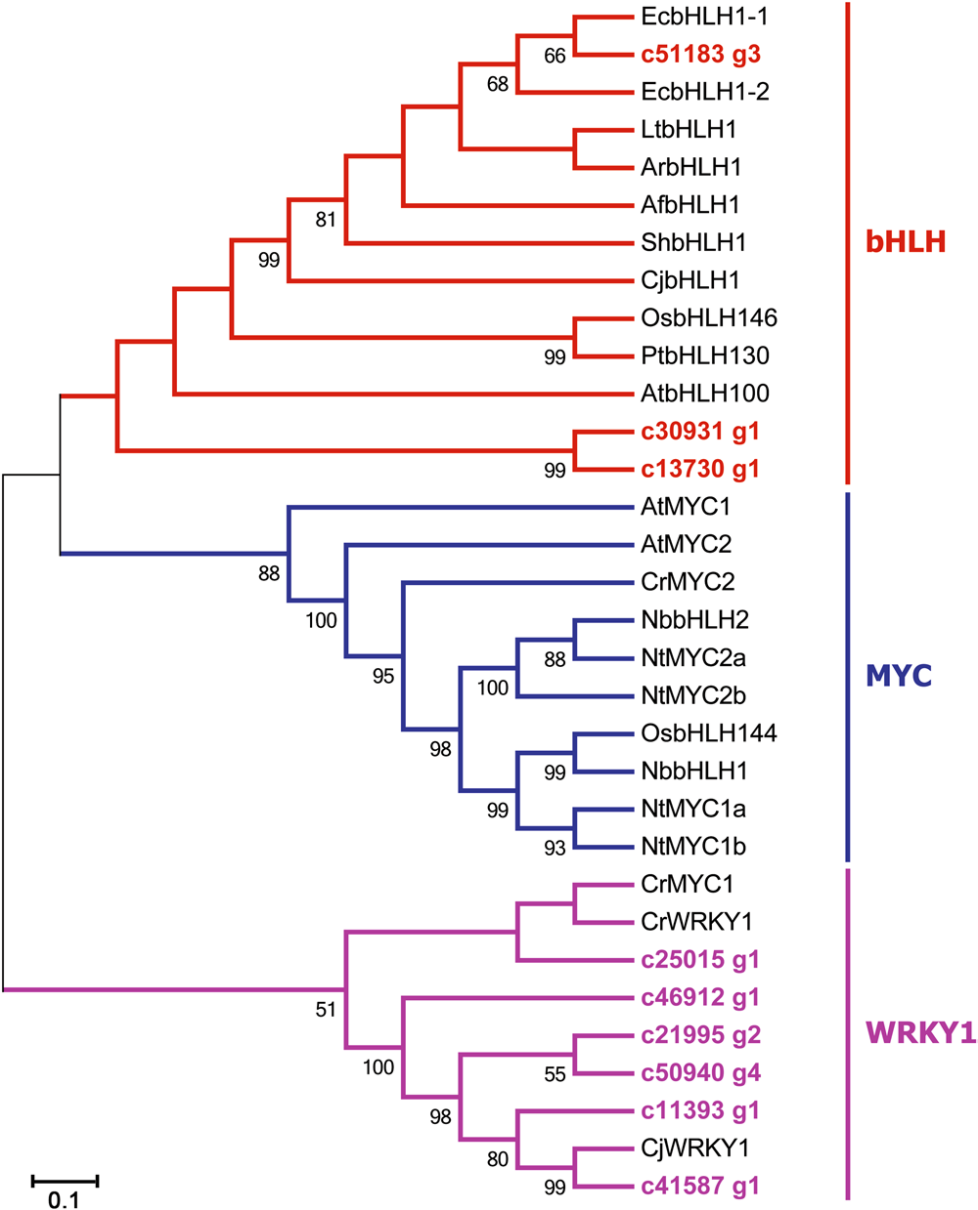
Phylogenetic analysis of TFs. Phylogenetic tree was constructed based on the deduced amino acid sequences for *D*. *scandens* TFs (bold letters) and other plant TFs involved in IQA biosynthesis. Accession Nos. are: Q39204, AtMYC2; Q8W2F1, AtMYC1; Q9ZVB5, AtbHLH100; AAQ14331, *Catharanthus roseus* CrMYC1; AAQ14332, CrMYC2; GQ859152, OsbHLH144; BAF14724, OsbHLH146; EEC73367, poplar PtbHLH130; EEE73911, *Nicotiana benthamiana* NbbHLH1; GQ859153, NbbHLH2; GQ859158, *N*. *tabacum* NtMYC1a; GQ859160, NtMYC2a; GQ859159, NtMYC1b; GQ859161, NtMYC2b; DT752478, *Aquilegia formosa* AfbHLH1; FD498024, *Liriodendron tulipifera* LtbHLH1; DT584473, *Saruma henryi* ShbHLH1; FD755492, *Aristolochia fimbriata* ArbHLH1; AB910896, EcbHLH1-1; AB910897, EcbHLH1-2; AB267401, CjWRKY1; AB564544, CjbHLH1; ADT82685, *C*. *roseus* CrWRKY1.

Transcription factors (TFs) play a crucial role in secondary metabolism by regulating expression of related genes at the transcriptional level to control the flux of secondary metabolites. So far, researches on TFs regulating IQAs biosynthesis have mainly focused on WRKY and basic helix-loop-helix (bHLH) families. In *C*. *japonica* cells, suppressing the expression of *CjWRKY1 *and *CjbHLH1* significantly decreased the expression of genes related to berberine biosynthesis^52–54^. Some Arabidopsis MYC2-type bHLH TF, such as *NbbHLH1/NbbHLH2/NtMYC2* and *CrMYC2*, have been reported to be involved in the regulation of nicotine biosynthesis in Nicotiana plants and terpenoid indole alkaloid biosynthesis in *Catharanthus roseus* ^55–58^. Recently, Yamada *et al*. (2015) also reported two non-AtMYC2-type genes (*EcbHLH1–1* and *EcbHLH1-2*) from *E*. *californica* were homologous to *CjbHLH1*, and the suppression of *EcbHLH1* genes, particularly *EcbHLH1-2*, resulted in down-regulated expression of some IQA biosynthetic enzyme genes and sanguinarine accumulation^59^. In the transcriptomic data of *D*. *scandens*, we identified 71 unigenes encoding bHLH TFs, and 52 unigenes encoding WRKY TFs (Supplementary Table S3). The phylogenetic relationship between TFs from *D*. *scandens* and TFs characterized from other plants was showed in Fig. 7. We identified three unigenes (c51183_g3, c30931_g1, and c13730_g1) belonged to bHLH, and c51183_g3 was closely homologous to EcbHLH1-1. Six unigenes (c25015_g1, c46912_g1, c21995_g2, c50940_g4, c11393_g1, and c41587_g1) were clustered with WRKY1, with c41587_g1 highly homologous to CjWRKY1, suggesting that they might be involved in regulation of IQAs biosynthesis in *D*. *scandens*. Characterizing the functions of these unigenes will help us to better understand the regulatory mechanism of IQAs biosynthesis.

## Conclusions

We firstly carried out transcriptomic analysis of *D*. *scandens* and obtained a total of 96,741 unigenes, which provide valuable genetic resource for this invaluable Chinese herb medicine. We further proposed the integrated biosynthetic pathways of isocorydine, corydine, glaucine and sinomenine in *D*. *scandens*. The identification of 67 unigenes and *in vitro* enzymatic characterization of one of them provide opportunities for the *de novo* production of active ingredients by microorganism engineering. In addition, identification and phylogenetic analysis of WRKY and bHLH TFs potentially involved in regulation of IQAs biosynthesis is of great importance to reveal molecular basis of IQAs biosynthesis pathways.

## Methods

### Plant materials

Wild seedlings of *D*. *scandens* collected from Weishan County, Yunnan Province, southwest China (25°23′N, 100°33′E and altitude: 2900 m) were transplanted into the field for 1.5 years. The roots, leaves and stems were harvested separately, immediately frozen in liquid nitrogen, and stored at −80 °C until use.

### cDNA library construction, sequencing and *de novo* assembly

Total RNA was extracted from roots, leaves and stems using Trizol reagent (Invitrogen, New York, USA) following by purification with RNeasy MiniElute Cleanup Kit (Qiagen, Hilden, Germany), according to the manufacturers’ protocols. For mRNA library construction and deep sequencing, at least 20 μg of total RNA samples were prepared using the NEBNext^®^ Ultra™ RNA Library Prep Kit for Illumina sequencing on a Hiseq. 2000 platform. Three biological replications were performed for each tissue.

Raw reads were firstly transformed into clean reads by removing reads with sequencing adaptors, reads with frequency of unknown nucleotides above 5% and low-quality reads (containing more than 50% bases with Q-value ≤ 20) using a custom Perl script. Then, the clean reads were *de novo* assembled using the Trinity program (k-mer = 25, group pairs distance = 300) with default parameters^43^.

### Functional annotation and candidate gene prediction

For functional annotations, all unigenes were assessed by public databases, including NT, NR (http://www.ncbi.nlm.nih.gov/), SwissProt (http://www.expasy.ch/sprot) and KOG database (http://www.ncbi.nlm.nih.gov/COG/), using BLASTX (E-value < 10^−5^) and BLASTN (E-value < 10^−5^), respectively. The unigenes were also aligned to KOG and KEGG databases (http://www.genome.jp/kegg)^60^ using BLASTX with an E-value < 10^−10^. A Perl script was used to retrieve KEGG Orthology information from blast result and then established pathway associations between unigenes and databases. Based on the results of the NR database annotation, the Blast2GO program^61^ was used to obtain GO unigene annotations. Then, WEGO^62^ software was used to perform GO classification and draw a GO tree. Moreover, the conserved domains/families of the assembled unigenes encoding proteins were searched against the Pfam database (version 26.0)^63^using Pfam_Scan script.

The CDSs of all unigenes were predicted using BLSATX and ESTscan. The unigene sequences were searched against the NR, KOG, KEGG and SwissProt protein databases using BLASTX (E-value < 10^−5^). The best alignment results were used to determine the sequence direction of unigenes. Unigenes with sequences with matches in one database were not searched further. When a unigene was not aligned to any database, ESTScan^64^ was used to predict coding regions and determine sequence direction. To identify the TFs, all unigenes were searched against the PlnTFDB database^65^ using iTAK analysis tool (http://bioinfo.bti.cornell.edu/cgi-bin/itak/index.cgi)^66^.

### HPLC analysis

0.2 g dried powder of *D*. *scandens* roots, leaves and stems was respectively extracted with 50 mL of 1% hydrogen chloride −70% methanol mixed liquor for 60 min, and sonicated for 30 min. For determining main bioactive components of *D*. *scandens*, an Agilent 1260 HPLC system (Agilent Technologies, Santa Clara, CA, USA) was used. Chromatographic separation was performed on the chromatographic column Agilent Zorbar SB-C 18 (250 mm × 4.6 mm, 5 μm, Agilent Technologies) at a column temperature of 30 °C. The flow rate was fixed at 1 mL/min, and the mobile phase consisted of sodium dihydrogen phosphate-methanol (35:65, v/v) containing 0.1% sodium dodecyl sulphate (**A**) and acetonitrile (**B**). Separation was achieved using the following gradient system: 85% B at 0 min, 100% B at 10 min, and 100% B at 30 min. Detection was performed at 289 nm^67^. Authentic (*S*)-corytuberine, isocorydine, sinomenine and protopine were purchased from JK chemical (Beijing, China).

### Digital gene expression profiling

The high-quality reads were aligned to the assembled unigenes with the BWA program^68^. An RPKM value was calculated for each unigene in each tissue of *D*. *scandens*. The RPKMs of all annotated isoforms for the same gene were summed as the RPKM of that gene. Differential expression of unigenes was calculated with a threshold of P value < 0.001 and two-fold change.

### Phylogenetic analysis

Phylogenetic analysis was performed based on the deduced amino acid sequences of OMTs and TFs from *D*. *scandens* and other plants. All deduced amino acid sequences were aligned with Clustal X using the default parameters as described previously^69^: gap opening penalty, 10; gap extension penalty, 0.1; and delay divergent cutoff, 25%. The evolutionary distances were computed using MEGA5.10 with the Poisson correction method. For the phylogenetic analysis, a neighbor-joining tree was constructed using MEGA5.0. Bootstrap values obtained after 1000 replications are indicated on the branches. The scale represents 0.1 amino acid substitutions per site.

### Recombinant protein purification and enzyme activity assay

Full-length cDNA of *DsOMT* was obtained by PCR amplification using primers 5′- CATATGATGAATCACAAAGTGCATCATCAT-3′ (forward, with the added *Nde*I restriction site underlined) and 5′-TCTAGATTATTTGCAGAACTCCATGACCCA-3′ (reverse, with the added *Xba*I restriction site underlined), and cloned into the pCzn1 vector (Zoonbio Biotechnology, China). The vector was introduced into the *Escherichia coli* line Arctic-Express (Zoonbio Biotechnology, China) for protein expression. The expression of the recombinant protein was induced by 0.5 mM of IPTG at 11 °C for 8 h. The cells were harvested by centrifugation and resuspended in binding buffer, and the suspension was subsequently homogenized by 1 h of 200Wsonication (Vibra Cell VC 505 Sonicator; Sonics & Materials, Newtown, CT). Cell debris was subsequently removed with 10-min centrifugation at 12,000 rpm. After renaturation by 2 M urea, the protein was purified by Ni-IDA -Sepharose CL-6B (Spectrum Chemical Manufacturing, USA) under the manufacturer’s instructions. The purity of the His-tagged protein was determined by SDS-PAGE followed by Coomassie Brilliant Blue staining.

The standard enzyme assay for DsOMT activity was performed using a reaction mixture in 50 μl of 100 mM Gly-NaOH (pH 9.0), 25 mM sodium ascorbate, 100 μM SAM, 10% (v/v) glycerol, 1 mM *β*-mercaptoethanol, 100 μM potential alkaloid substrate, and 50 μg of purified recombinant enzyme. Assays were carried out at 37 °C for 2 h and terminated by adding 200 μL of 1 M NaHCO_3_. Products were identified by HPLC as described above. Control was performed with denatured purified His-tagged proteins prepared by boiling in water for 20 min.

## Electronic supplementary material

Supplemental Figures

Supplementary Tables
