# Supplementary material for: Identification of candidate genes involved in isoquinoline alkaloids biosynthesis in Dactylicapnos scandens by transcriptome analysis
Source: Sci Rep. 2017 Aug 22;7:9119. doi: 10.1038/s41598-017-08672-w (PMC5567367; doi:10.1038/s41598-017-08672-w)
Supplement: Supplementary file 1 — Supplemental Figures [file 41598_2017_8672_MOESM1_ESM.doc]

**Supplementary information for following article**

**Identification of candidate genes involved in isoquinoline alkaloids biosynthesis in *Dactylicapnos scandens* by transcriptome analysis**

Si-Mei He^1^, Wan-Ling Song^1^, Kun Cong^1^, Xiao Wang^2, 5^, Yang Dong^3^, Jing Cai^4^, Jia-Jin Zhang^1^, Guang-Hui Zhang^1^, Jian-Li Yang^6^, Sheng-Chao Yang^1*^, Wei Fan^1*^

^1^State Key Laboratory of Conservation and Utilization of Bio-resources in Yunnan, National& Local Joint Engineering Research Center on Gemplasm Utilization & Innovation of Chinese Medicinal Materials in Southwest China, Yunnan Agricultural University, Kunming, China

^2^State Key Laboratory of Genetic Resources and Evolution, Kunming Institute of Zoology, Chinese Academy of Sciences, Kunming, China. 32 East Jiaochang Road, Kunming, 650223, Yunnan, People’s Republic of China

^3^Province Key Laboratory, Biological Big Data College, Yunan Agricultural University, Kunming, 650201, Yunnan, People’s Republic of China

^4^State Key Laboratory of Quality Research in Chinese Medicine, Institute of Chinese Medical Sciences, University of Macau, Avenida da Universidade, Taipa, Macau SAR, People’s Republic of China

^5^Graduate School of the Chinese Academy of Sciences, Beijing 100049, China

^6^State Key Laboratory of Plant Physiology and Biochemistry, College of Life Sciences, Zhejiang University, Hangzhou 310058, China

*Corresponding authors: Sheng-Chao Yang (shengchaoyang@163.com); Wei Fan (fanwei1128@aliyun.com)


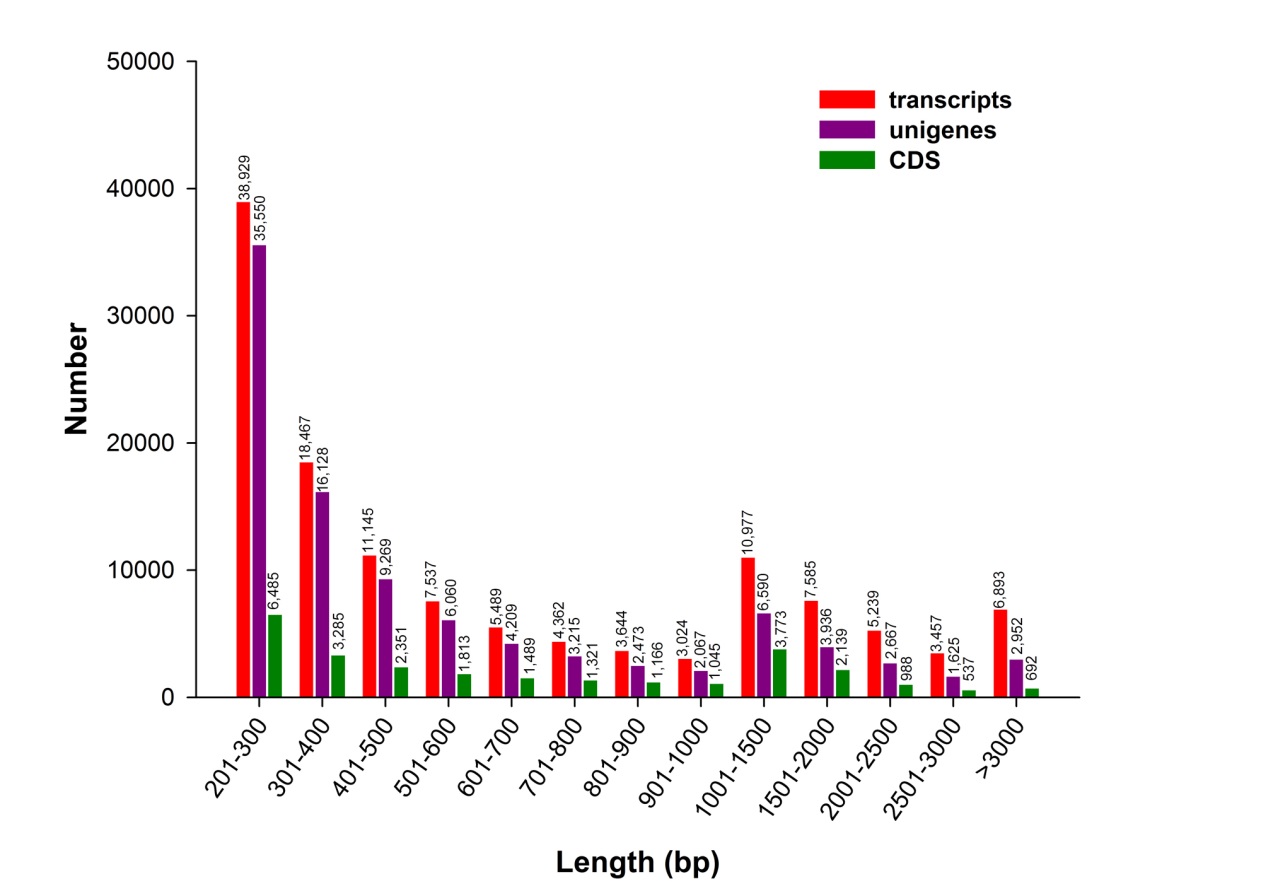


**Supplementary** **Figure 1 Length distribution of transcript, unigenes and CDSs.**


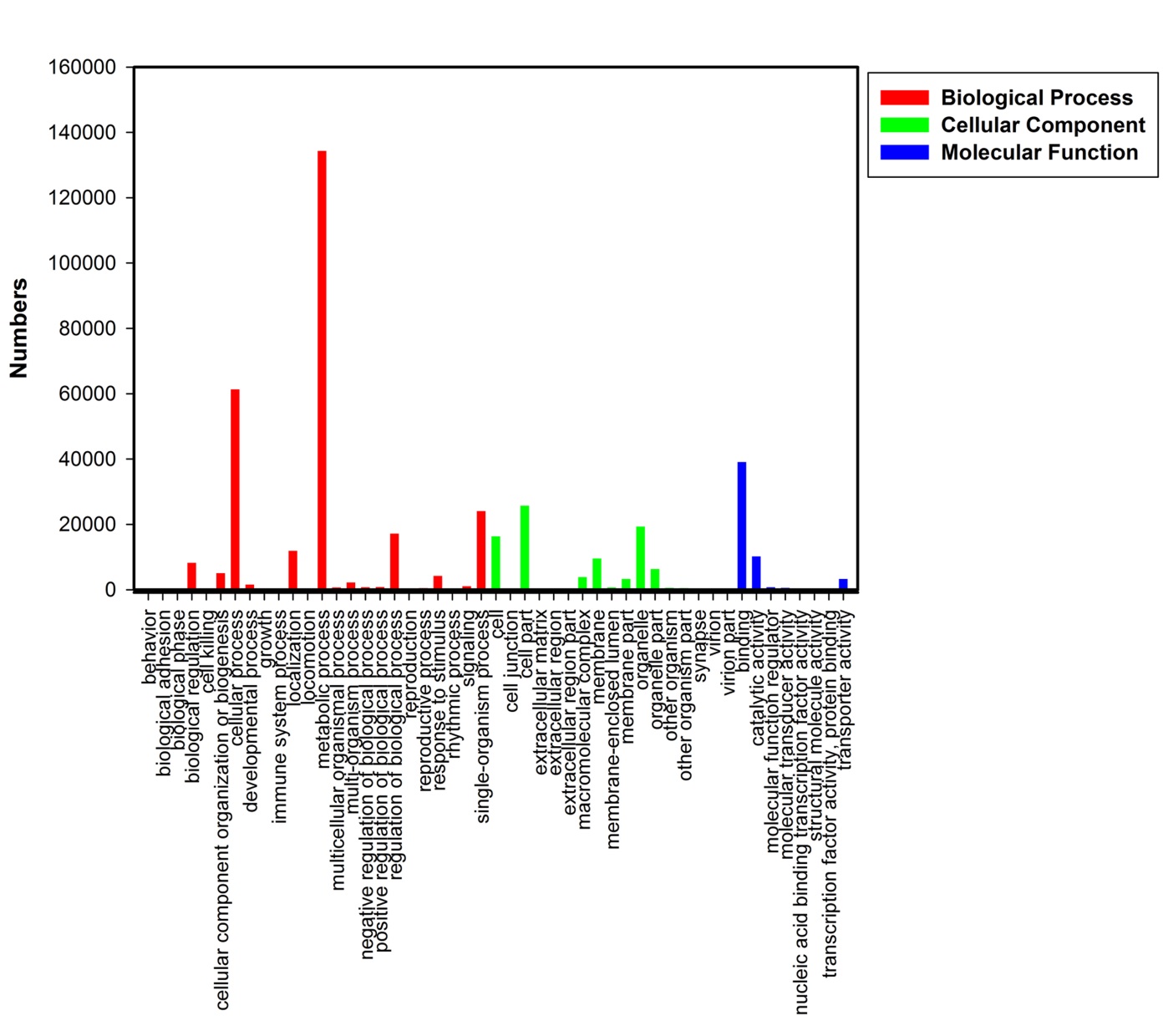
 **Supplementary** **Figure 2 Gene Ontology classification of assembled unigenes.**

**
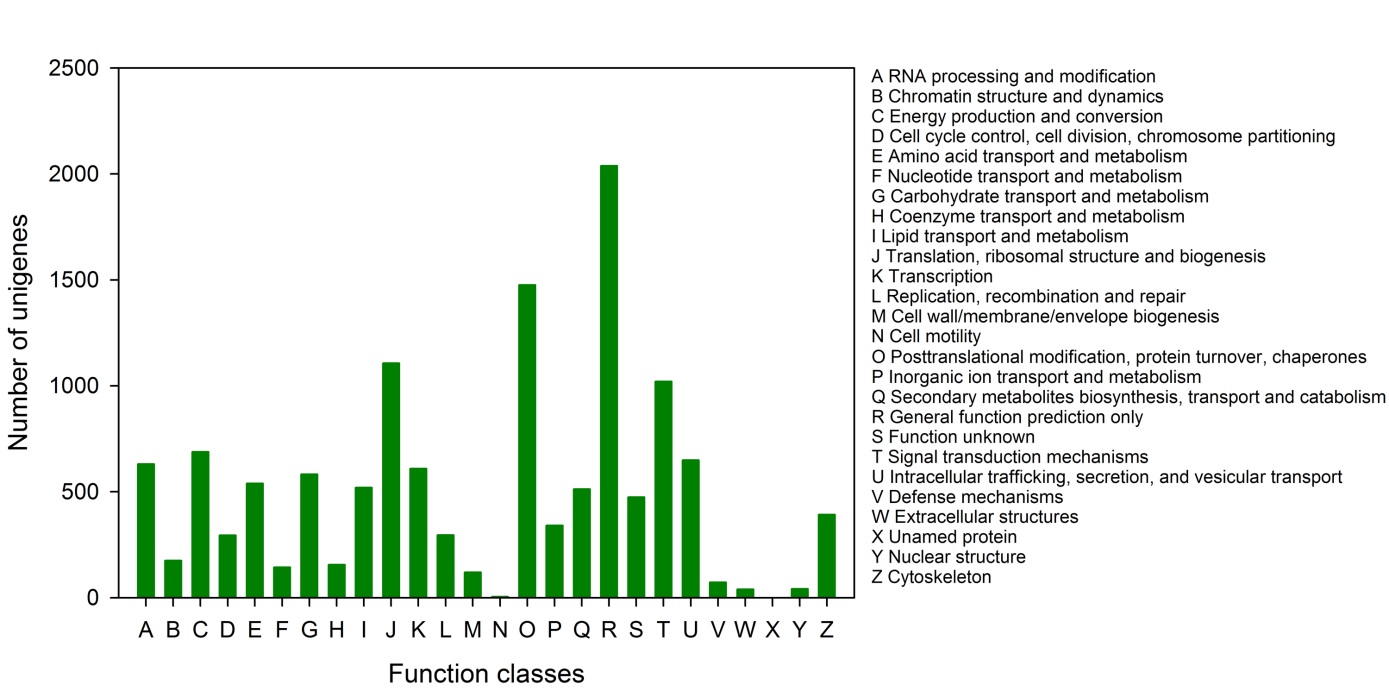
 Supplementary** **Figure 3 KOG function classification of *D. scandens* unigenes.**


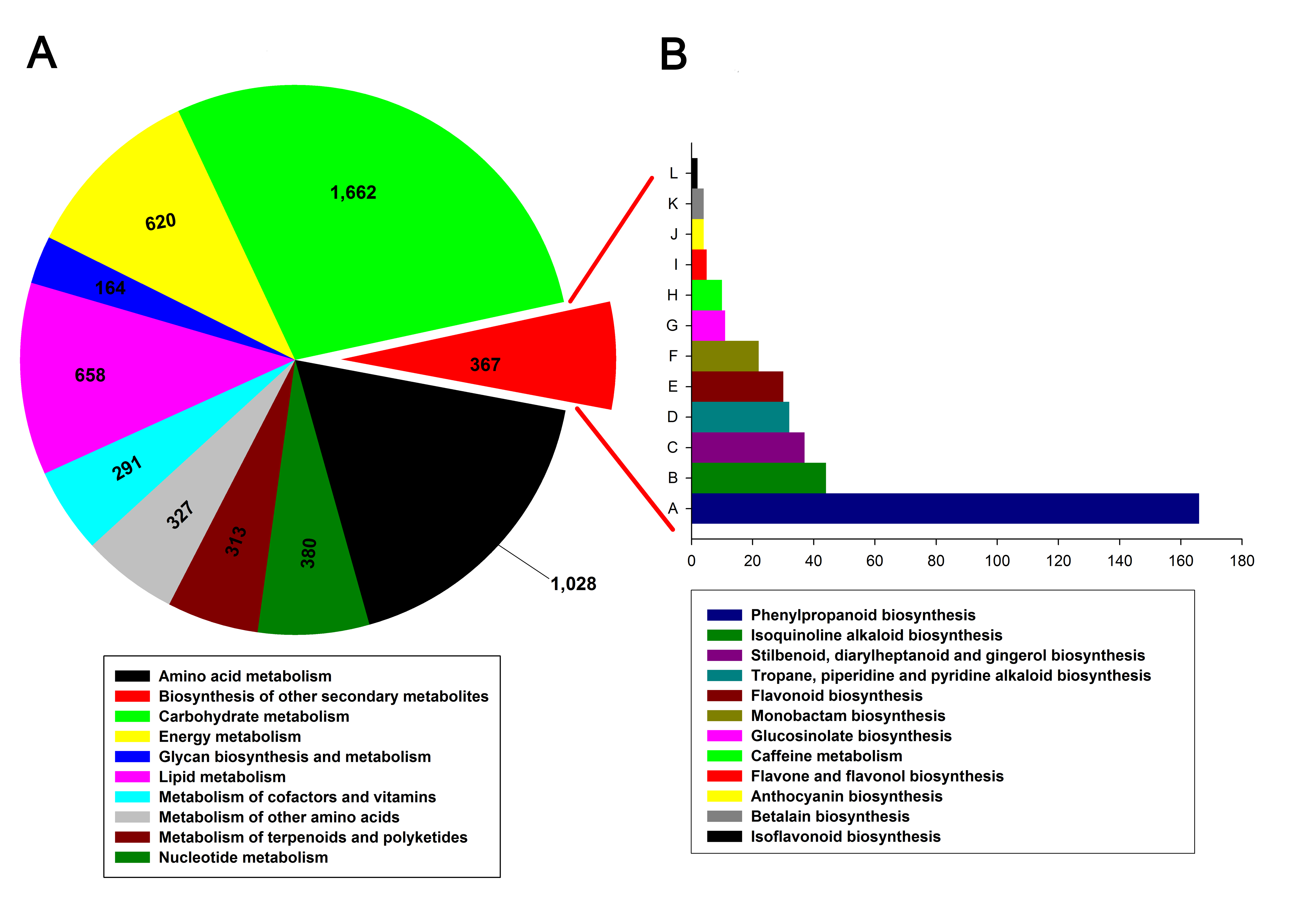


**Supplementary** **Figure 4 Pathway assignment based on KEGG.** (A) Classification based on metabolism categories; (B) classification based on biosynthesis of secondary metabolites.
